# Remarks on the Tractus Intestinalis

**Published:** 1903-03

**Authors:** Byron Robinson

**Affiliations:** Chicago


For Texas Medical Journal.

### REMARKS ON THE TRACTUS INTESTINALIS.

BY BYRON ROBINSON, B. S., M. D., CHICAGO.

**Function.**

- Secretion.

- Absorption.

- Peristalsis.

**Blood supply.**

- Gastrium—Cœliac axis.

- Enteron—superior mesenteric

- colon—Inferior mesenteric.

**Segments.**

- Gastrium—stomach.

- Enteron—

  - Duodenum.

  - Jejunum.

  - Ilium.

- Colon.

  - Appendix, 3½ in.

  - Cœcum, 1x2½ in.

  - Right colon, 7 in.

  - Transverse colon, 22 in.

  - Left colon, 8 in.

  - Sigmoid, 19 in.

  - Rectum, 4 in.

  - Anus, 1 in.

**Nerve supply.**

- Sympathetic.

- Cerebral pneumogastric.

- Spinal—sacral

**Tunics, coats.**

- Tunica mucosa.

- Tunica submucosa.

- Tunica muscularis

- Tunica serosa.

**Accessory Glands**

- 1—Salivary.

- 2—Hepar.

- 3—Pancreas.

- 4—Spleen.

**Sphincters.**

- Cardiac.

- Pylorus.

- Ileocacal.

- Cæco appendicular.

- Anal

  - Internal.

  - External.

**General Disease.**

- 1—Inflammation, endointestinalis.

- 2—Constipation (defective).

  - Peristalsis.

  - Sensation.

  - Secretion.

  - Absorption

- 3—Malignancy.

- 4 Strictures.

  - Sphincters and flexures.

**Flexures.**

- Duodeno, Jejunalis.

- Hepatic.

- Splenic.

- Signoid

**Function of Sphincters.**

- *a*—To guard an orifice.

- *b*—To contract and relax

- *c*—To retard food.

**Characteristics of a sphincter.**

- *a*—Congestion and decongestion.

- *b*—It has a high blood supply.

- *c*—It has a delicate nerve supply.

- *d*—It has a large lymph supply.

**The ætiology and method of formation of stricture in sphincters and flexures.**

- 1—Abrasion of the mucosa over the structure.

- 2—A wound or atria of infection.

- 3—Formation in wound of proliferating connective tissue.

- 4—Contraction of the connective tissue—(stricture).

**Microbes of interest:**

- Bacillus coli communis.

- Bacillus tuberculosis.

The tunica serosa is a lymph coat, and is frequently diseased (peritonitis) by infective germs, which arrive at the tunica serosa or mucosa, or from tunica mucosa or from the side of the peritoneum (through instrumentation or perforation). Inflammation of the tunica serosa (peritonitis) causes paresis of the tunica muscularis (myositis) in the tractus intestinalis and disproportionate secretion, induces fermentation and consequent formation of gas, ending in tympanitis.

The appendix is the weakest segment of the tractus intestinalis, and produces the most dangerous and treacherous abdominal disease. Peritonitis is the fell destroyer of man, and he who brings further means to prevent and remove it will be a race benefactor like Jenner, Semmelweis, Pasteur, Lister, Tait and Fenger.

Inflammation of the peritoneum is one of the most potent factors in producing appendicitis. The peri appendicular peritoneum becomes inflamed, exudates arise and contractions result which compromise the lumen of the appendix as well as its blood and lymph supply, traumatizes its nerve periphery, rendering its nourishment defective, whence the crisis of appendicular obstruction rapidly brings on perforation.

The tunica muscularis is not frequently diseased (myositis) because the finely woven, powerful fascial membrane, submucosa, prevent germs from passing through it.

The tunica mucosa of the tractus intestinalis is frequently diseased and known as catarrh, or even solution of continuity, ulceration, which consists in a partial degeneration of the epithelia. Its characteristics are hypersecretion of mucus.

A full intestine is always in paristalis or rhythmical motion. An empty intestine is a quiet one. Hence, material for food should be of such a nature as to leave a large quantity of residue to resist muscular action. The tractus intestinalis should be evacuated in abdominal section for space to operate and to avoid visceral trauma. In splanchnoptosis the tractus intestinalis (a) passes distalward, following (b) the relaxed abdominal walls and also (c), gastroduodenal dilatation occurs from pressure of the superior mesenteric vein, artery and nerve on the transverse segment of the duodenum.

The tractus intestinalis is subject to reflex irritation from diseases in the other abdominal visceral tracts, viz.: tractus genitalis, tractus urinarius and tractus peritonei.

The mesenteries are not made for mechanical support, but for neuro-vascular visceral pedicles. The tractus intestinalis is suspended by the mesentery in the abdominal cavity like a man in water with a cord tied to him. The mesentery is not for mechanical support. It is a neurovascular visceral pedicle. The great supporter of the tractus intestinalis is the abdominal walls, besides the abdominal walls (including the thorax and pelvic diaphragm) massage the tractus intestinalis, regulate its lymph and blood flow, secretion, absorption and peristalsis.

The gastrium is a food reservoir (with a rhythm when containing food). The enteron is the business or absorption and secretive portion of the tractus intestinalis (with about a six-hour rhythm). The colon is a fœcal reservoir (with a daily rhythm). The duodenum is the most important segment of the tractus intestinalis, as it receives the ductus hepaticus and ductus pancreaticus. The appendix is the weakest and most dangerous segment of the tractus intestinalis on account of its atrophy and consequent nonresistance. The tractus intestinalis is longer than the body, and hence it must assume a coiled state. It was origianlly made for a quadruped, hence in the upright position (man, ape) it suffers in its circulation nerve, periphery and dorsal attachments. The tractus intestinalis suffers from peritoneal adhesions from muscular trauma, especially where it rests on and crosses muscles, *e. g.*, the psoæ, the pillars of the diaphragm, the diaphragm, the ventral and lateral abdominal muscles. Consequently, at the above points of muscular trauma the segments of the tractus intestinalis are compromised in circulation, peristalsis and nerve periphery by peritoneal adhesions which disturb mechanism structure and function, physiology.

### GENERAL TREATMENT OF THE TRACTUS INTESTINALIS.

In man the tractus intestinalis should be evacuated once daily. The chief disease of the tractus intestinalis is constipation, which consists in a disturbed peristalsis, sensation, absorption and secretion. Woman suffers more from constipation than man because of her sedentary life, and also because in child-bearing period woman's tractus genitalis is more ill than that of man, hence her tractus intestinalis suffers more from reflex causes. One of the chief causes of constipation is lack of ample fluids at regular intervals. For many years I have adopted a systematic drainage treatment for the tractus intestinalis. Its success on large numbers of patients in hospital and private treatment is sufficient to continue it. The best drain for the tractus intestinalis is one-half normal salt solution, eight ounces every two hours for six times daily. I have a NaCl tablet made so that one dissolved in a glass full of water or eight ounces will make one-half normal salt solution. To this I add what I term an alkali tablet, consisting of socratine aloes, grs. 1/8; fl. ext. cascara sagrada, grs. 1-40; $NaHCO_3$, grs. I; $KHCO_3$, grs. 1/3; $MgSO_4$, grs. 2.

The advice given to the patient is to take a glass full of water every two hours (better hot) with a NaCl tablet dissolved in it, also to swallow dry one-half to one alkali tablet immediately before drinking the eight ounces of half normal salt solution. Following this method for a few months will allow the omission of the alkali tablet. The half normal salt solution eight ounces every two hours should be continued, for a tractus intestinalis once suffered from constipation may become re-attacked at almost any time. In my practice this method has made hundreds a year have regular evacuations. I have the NaCl tablets made in large quantities, as well as the alkali tablets. They are each of different size and color and easy to dispense, as well as of convenient use to patients.

A notable feature in the treatment is that one-half normal salt solution changes the urine in four to six days from dark mahogany color to a clear solution, resembling spring water. Try it.

